# Facial affect recognition in first-episode psychosis is impaired but not associated with psychotic symptoms^☆^

**DOI:** 10.1016/j.heliyon.2022.e10424

**Published:** 2022-08-27

**Authors:** Cornelia Larsson, Maria Lee, Tobias Lundgren, Sophie Erhardt, Carl M. Sellgren, Simon Cervenka, Jacqueline Borg, Sven Bölte, Helena Fatouros-Bergman

**Affiliations:** a Centre for Psychiatry Research, Department of Clinical Neuroscience, Karolinska Institutet, & Stockholm Health Care Services, Region Stockholm, Stockholm, Sweden; b Department of Physiology and Pharmacology, Karolinska Institutet, Stockholm, Sweden; c Department of Medical Sciences, Psychiatry, Uppsala University, Uppsala, Sweden; d Center of Neurodevelopmental Disorders (KIND), Centre for Psychiatry Research; Department of Women's and Children's Health, Karolinska Institutet & Stockholm Health Care Services, Region Stockholm, Stockholm, Sweden; e Child and Adolescent Psychiatry, Stockholm Health Care Services, Region Stockholm, Stockholm, Sweden; f Curtin Autism Research Group, Curtin School of Allied Health, Curtin University, Perth, Australia

**Keywords:** First episode psychosis (FEP), Facial affect recognition (FAR), Cognition, Emotion recognition, Social cognition, Antipsychotic drug-naïve

## Abstract

**Introduction:**

Social dysfunction is a key feature of psychotic disorders such as schizophrenia linked to disability. Less is known about social functioning in the early stages of the disorder and if there is an association to psychotic symptoms.

**Aims:**

Investigate if antipsychotic drug-naïve or briefly medicated individuals with first-episode psychosis (FEP), have impaired facial affect recognition (FAR) compared to control participants and if psychotic symptoms are associated with the FAR ability.

**Method:**

Individuals with FEP (n = 67) and control participants (n = 51) performed a computer-aided FAR task on basic emotions. Psychotic symptoms were assessed with the Positive and Negative Syndrome Scale (PANSS). Group performances were compared using age and gender as covariates. The associations between FAR and performance on the subscales of PANSS were analyzed.

**Results:**

Compared to control participants, individuals with FEP were impaired in general FAR (Beta = -2.04 [95 % conf: -3.75/-1.62], p < 0.001) and FAR of negative emotions (Beta = -1.74 [95 % conf: -3.08/-1.22], p < 0.001), driven by difficulties in recognition of anger and disgust. In both groups, there was a pattern of mistaking negative emotions for other negative emotions. There were no significant group differences in FAR of happiness. No significant associations between FAR and psychotic symptoms were observed.

**Discussion:**

The results indicate that FAR, an underlying mechanism of social functioning is impaired early in the course of psychotic disorders. Current findings do not support the hypothesis that misinterpretation of facial expressions in individuals with FEP underlies or contributes to symptoms of psychosis.

## Highlights

• Impaired affect recognition (FAR) present already in first episode psychosis (FEP).
• Preserved FAR of happiness found in individuals with FEP.
• Individuals with FEP mainly have impaired FAR of negative affects.
• Both FEP and controls mainly mistook negative affects for other negative affects.
• Psychotic symptoms are not associated with FAR in individuals with FEP.

## 1 Introduction

Social functioning is often impaired in individuals with psychotic disorders such as schizophrenia. Affected individuals report fewer social contacts and activities (Velthorst et al., 2017), a lack of romantic relationships (Harvey, 2014), and lower quality of life (Velthorst et al., 2017). Social cognition, the ability to process social information and respond appropriately, is a key component in social functioning (Green, 2016). One aspect of social cognition is the ability to accurately recognize emotional states in others, where one of several capabilities required (Hassin et al., 2013; Juckel et al., 2018; Newen et al., 2015) is facial affect recognition (FAR). This ability is important in many aspects of daily life, and deficits in this domain have been associated with functional disability (Green, 2016), limited social skills (Meyer and Kurtz, 2009), and poorer community outcomes in psychotic disorders and schizophrenia (Fett et al., 2011).

Lower scores on tests operationalizing FAR, mostly using emotion labeling, are reported in individuals with schizophrenia (Chan et al., 2010; Kohler et al., 2010; Mote and Kring, 2016; Savla et al., 2013). To discern whether this impairment is present already at illness onset or attributed to effects of chronic illness or pharmacological treatment, it is essential to study antipsychotic drug naïve individuals with first-episode psychosis (FEP). Findings in antipsychotic medicated FEP patients are contradicting. Some studies have reported effect sizes of equal magnitude as in individuals with long-term schizophrenia (Barkl et al., 2014) whereas other studies report less impaired FAR, suggesting deterioration over time (Romero-Ferreiro et al., 2016; Thompson et al., 2012).

It is still unclear whether FAR deficits are general or emotion-specific in FEP. To date, relatively few studies have investigated this and findings have been mixed. A pattern that has been suggested (Barkl et al., 2014) is a more pronounced deficit in recognizing negative affects (Wilmot et al., 2012) such as anger, sadness (Romero-Ferreiro et al., 2016), fear, and disgust (Leung et al., 2011), whereas FAR of happiness (Comparelli et al., 2012; Leung et al., 2011) and neutral affect (Barkl et al., 2014; Catalan et al., 2016; Healey et al., 2016) is more preserved in FEP. However, there are contradicting results (Barkl et al., 2014; Healey et al., 2016) and an additional lack of studies on drug naïve or briefly medicated patients with FEP. A related topic is the type of errors that individuals with FEP make when they are mistaken. To our knowledge, such data has been only sparely reported before (Catalan et al., 2016; Tripoli et al., 2022) and could offer insights into the perceptual and cognitive processes underlying FAR deficits.

Apart from leading to impairment in social functioning, misinterpretation of facial expressions has been hypothesized to contribute to psychotic symptoms, such as delusions and hallucinations (Hillmann et al., 2018). Building on this line of thought, psychological interventions for psychotic experiences include developing a more flexible interpretation of the intentions of others (Kingdon and Turkington, 2008; Moritz and Woodward, 2021). Moreover, FAR deficits could underlie negative symptoms such as decreased interest or lack of motivation. Some earlier studies found associations between psychotic symptoms and impaired FAR in individuals with schizophrenia and FEP, both for negative (Healey et al., 2016; Kohler et al., 2010; Schneider et al., 1995) and positive symptoms (Bonfils et al., 2019; Healey et al., 2016; Kohler et al., 2010; Won et al., 2019). However, in a review of individuals with FEP the results regarding associations between FAR and psychotic symptoms were mixed, with approximately half of the studies reporting a significant association (Healey et al., 2016).

Taken together, there is a need for more research on facial affect recognition, specifically in antipsychotic drug naïve patients in the early phase of psychotic illness, including the relationship between FAR and psychotic symptoms (Healey et al., 2016). We therefore aimed to compare FAR ability of antipsychotic-naïve or briefly medicated individuals with FEP to control subjects and examine the associations between FAR ability and psychotic symptoms. Our hypotheses were that FEP participants in this very early stage of illness would show impairments in general FAR ability, FAR of negative and positive affects, and that FAR ability would be associated with negative and positive psychotic symptoms.

## 2 Method

The present study was approved by the Regional Ethics Committee in Stockholm (diary number: 2010/879-31-1). After receiving a full description of the study, participants provided written informed consent, in accordance with the Declaration of Helsinki.

### 2.1 Participants

Individuals with FEP and control subjects were recruited as part of the Karolinska Schizophrenia Project (KaSP); a multidisciplinary research group that enrolls individuals who seek health care with psychotic symptoms for the first time in the greater Stockholm area. Control participants were healthy subjects recruited as a convenient sample via advertisement on an online platform listing research projects enrolling controls. Since controls were recruited to several subprojects within KaSP, the proportion of controls varied, a subsample was also matched to patients based on age and gender. All participants, both patients and controls, received monetary compensation. Although the recruitment to KaSP is still ongoing, included participants in this study were recruited 2011–2018. Inclusion criteria for participants with FEP were meeting the criteria for a psychotic disorder as defined using the DSM-IV, assessed by an MD using the Structured Clinical Interview for DSM-IV (SCID-I). Half of the FEP participants were antipsychotic naïve and the other half had less than four weeks of exposure to antipsychotic medication. All patients underwent clinical characterization including the Global Assessment of Functioning (GAF) and Clinical Global Impression (CGI), to facilitate comparisons with other samples regarding illness severity and level of disability; as well as the Positive and Negative Syndrome Scale (PANSS) to assess positive, negative and general psychotic symptoms (Kay et al., 1987). Exclusion criteria for participants with FEP and controls were current,- or history of abuse of alcohol or illegal drugs (as assessed using The Alcohol Use Disorders Identification Test [AUDIT] and Drug Use Disorders Identification Test [DUDIT], severe somatic illness or neurological disorder (ruled out through medical history, clinical examination, laboratory tests, and brain magnetic resonance imaging). Participants with FEP were only included if they had less than four weeks of exposure to antipsychotic medication. Additional exclusion criteria for control participants were previous or current psychiatric illness assessed by Mini International Neuropsychiatric Interview, lifetime use of anti-psychotics, or first-degree relatives with psychotic illness.

As part of KaSP, participants with FEP and control participants underwent several examinations and clinical assessments within 1–2 weeks of enrollment. The mean time between PANSS assessment and FAR testing was 8,3 days (SD 10,2). In the KaSP cohort, 76 individuals with FEP and 53 controls completed the FAR task. Out of these, a total of 8 individuals with FEP and 2 controls were excluded due to; incomplete data (5 FEP, 1 control), somatic or neurologic disorder (3 FEP), and drug use (1 control). After the exclusion of one (1 FEP) additional statistical outlier a total of 118 participants (67 FEP, 51 controls) were included in the study.

### 2.2 FAR task

The Frankfurt Test and Training of Facial Affect Recognition 2^nd^ Edition (FEFA-2) is a standardized computer program to assess and develop basic emotion identification that has been utilized in a multitude of previous studies (Bölte et al., 2006, 2015; Bölte and Poustka, 2003; Kuusikko et al., 2009) and has demonstrated good psychometric properties (Bölte et al., 2002; Bölte, 2003). FEFA-2 presents black and white images of faces displaying one of the seven basic emotional states (happiness, sadness, fear, disgust, anger, surprise, neutral) using the emotion conception by Ekman (Ekman and Friesen, 1976). Participants were instructed to pick one of the seven emotions per image (49 items total) within 15 s (choices after this time limit were scored as incorrect). Based on the normalization of FEFA only one affect was deemed correct for the majority of images (referred to as pure, k = 34) while two different responses were considered to be a correct answer for a subset of pictures (referred to as “mixed”, e.g., happy surprise, k = 15). For all primary analyses, the “pure” category was used. The “mixed” category could be considered a proxy for subtle emotional expression. All participants were presented with the same images in the same order. Affects in FEFA-2 were presented an unequal number of times; Happiness 7 times, Sadness 6 times, Anger and Disgust 5 times, Surprise and Neutral 4 times, and Fear was displayed 3 times. One point was scored for every correct choice and total raw scores for “pure” emotions (min 0 to max 34), as well as a score for negative (anger + disgust + fear + sadness scores: min 0 to max 19) and positive FAR (happiness score: min 0 to max 7), were used for all analyses.

### 2.3 Statistics

The statistical analysis was pre-registered on Open Science Framework (osf.io) and can be found at: https://doi.org/10.17605/OSF.IO/MURWS. Data was examined in a confirmatory and an exploratory part. All calculations were performed in the software R.

#### 2.3.1 Power analysis

A power analysis was conducted with the R “*pwr*” package as part of the pre-registration, using effect sizes of group differences and correlations between FAR scores and PANSS reported in previous studies. Given our sample size and the aim of being able to detect a group difference of a medium to large effect size (approx. Cohen's d = 0.7) and correlation of moderate magnitude (considered to be an r = 0.5), we estimated that we were able to perform 5 group comparisons and 6 correlations with 80% power.

#### 2.3.2 Outliers

Outliers were defined as data points +/- 3.3 standardized residuals from the mean on the FEFA-2 test (FEFA Total). Three individuals met this criterion but none had a Cook's distance close to 1, or an overly large leverage on the model [considered to be over 0.099 (3∗(k+1/n))]. However, when examining the responses and source files from these individuals, only the validity of one person's responses was questionable [standardized residual (−4.98)]. This person was perceived as lethargic and alternated only between the responses Neutral and Happiness throughout the test. Therefore, this individual was excluded from all analyses while the other outliers were included.

#### 2.3.3 Confirmatory analyses

In the confirmatory part, the hypothesis that individuals with FEP and control participants differ in their FAR ability was tested. Group differences were examined in three FAR categories: FEFA-2 Total score (all “pure” emotions), FEFA-2 Negative affect (anger + disgust + fear + sadness), and FEFA-2 Positive affect (happiness). Age was included in the analysis as age has been shown to affect FAR (Hayes et al., 2020). Gender was also included in the model as gender distribution differed in the two groups. Group differences were tested using an ANCOVA, computed as a linear model in R with the formula: FAR ∼ FEP/control group + Age + Gender. Since problems with normality of residuals and homogeneity of variances were found in some cases robust versions of the ANCOVA for parameter estimates and bootstrapping were used to compute the confidence intervals and significance tests for our models. Estimates and p-values from the robust versions of the ANCOVA were very similar to the original non-robust model. Throughout the results, robust estimates and bootstrapped (with 1000 iterations) confidence intervals and p-values are presented. Estimates and p-values from the original model were very similar.

Furthermore, we aimed to test if FAR ability, in individuals with FEP, was correlated to their level of psychotic symptoms, measured by PANSS. Correlations were examined between FAR (General, Positive, Negative) and PANSS (Positive, Negative), yielding in a total of 6 analyses. Since some of the FEFA-2 categories being tested were not normally distributed and included several tied ranks, Kendall's tau partial correlation was used with age as a covariate.

We also examined which emotions the participants picked when they were mistaken, for every affect separately. The incorrect answers were summed up and presented as proportions for individuals with FEP and control participants respectively.

#### 2.3.4 Exploratory analyses

Individuals with FEP and control subjects were compared on all separate “pure” emotions tested by FEFA-2. We explored whether individuals with FEP and controls differed in their ability to accurately recognize the “mixed” category of FEFA-2, using the same linear model setup as for the confirmatory analyses above. The FEP group's performance in the “mixed” category was also correlated to their PANSS Positive and PANSS Negative scores. Furthermore, we hypothesized that positive symptoms of psychosis, such as delusions and hallucinations could be created or reinforced by impaired FAR and misinterpretation of other's emotions. Therefore, correlation analyses were performed between PANSS items Delusions and Hallucinations and FEFA-2 Total and FEFA Negative scores to explore this relationship.

Finally, we explored differences in FEFA-2 performance between the medicated subgroup, the drug naïve subgroup, and the controls.

#### 2.3.5 Correction for multiple comparisons

Given that both the FEFA scores (r = 0.07 to r = 0.94) and PANSS subscales were intercorrelated (r = 0.25), a Bonferroni correction for multiple comparisons was considered too conservative. Alpha levels for the confirmatory analyses were corrected by estimating the effective number of tests (Meff). This approach considers the correlations among the variables being tested while maintaining a consistent family-wise error rate of 5 % (Derringer, 2018). The group comparison between individuals with FEP and controls was considered one family of tests, while the correlation analyses in the FEP group were considered a different family of tests. This approach yielded a significance threshold of p = 0.021 for the comparison of groups and p = 0.011 for correlation analyses.

All statistical analyses were performed using the statistical software R version 3.6.2 (Team, 2020).

## 3 Results

Individuals with FEP and control participants did not differ in terms of age, gender, or years of education. Data on demographic variables are presented in Table 1.

**Table 1:** Demographic and clinical data, mean (standard deviation).

	FEP	Controls	X^2^	t-value	df	p-value
	n = 67	n = 51				
Gender (male/female)	41/26	26/27	1.77		1	0.184
Age	27.8 (6.9)	26.6 (5.7)		1.07	117.85	0.287
Education in years	14.1 (3.2)	14.8 (2.2)		-1.26	108.47	0.210
	n = 62	n = 52				
Duration of illness (months) n = 54	11.1 (14.9)					
	Median = 5					
	1^st^ quartile: 2					
	3^rd^ quartile: 12					
	Interquartile range = 10					
	(min = 0, max 84)					
PANSS						
Positive	18.4 (5.6)					
Negative	16.2 (6.3)					
General	36.8 (9.90)					
Total	71.4 (17.5)					
Status of antipsychotic medication						
Medicated	34					
1^st^/2^nd^ generation	3/30					
Drug naïve	33					
Diagnosis (DSM IV)						
Schizophrenia (295.xx)	22					
Schizophreniform Disorder (295.4)	21					
Psychosis NOS (295.4)	16					
Brief psychotic Episode (298.8)	3					
Delusional Disorder (297.1)	3					
Schizoaffective Disorder (295.7)	2					
Level of functioning						
CGI	4.4 (1.2)					
GAF (n = 66)	41.7 (12.4)					

### 3.1 Confirmatory analyses

**Figure 1:**
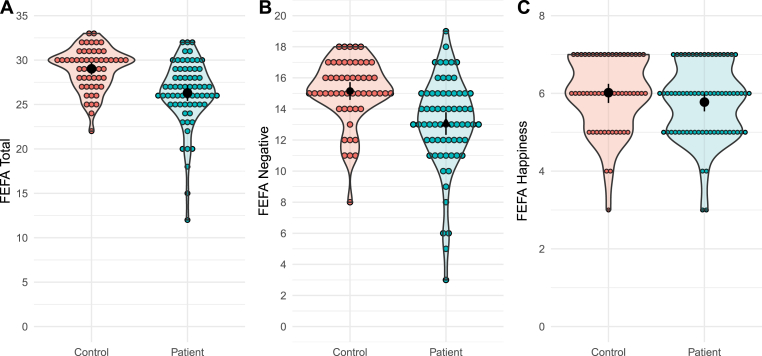
FEFA-2 scores for controls in red and individuals with FEP in blue.

The ANCOVA of FEFA-2 performance between groups revealed that FEP participants performed significantly lower on the FEFA-2 total (Beta = -2.04 [95 % conf: -3.75/-1.62], bootstrapped p < 0.001, Cohen's *d* = 0.84) and FEFA-2 negative emotions (Beta = -1.74 [95 % conf: -3.08/-1.22], bootstrapped p < 0.001, Cohen's *d* = 0.79), with FEP scoring an average of 2 (FEFA-2 total) and 1.7 points (FEFA-2 negative) lower than controls. No significant effect of group was detected when comparing performance on FEFA-2 Happiness (Beta = -0.20 [95 % conf: -0.58/0.16], bootstrapped p = 0.273, Cohen's *d* = 0.19) (see Figure 1).

Data for FEFA Total (A), FEFA Negative (B), and FEFA Happiness (C). Black-filled circles represent the group mean, with a 95 % confidence interval indicated by the black line.

In all participants, errors were most frequently observed for negative emotions, which were mistaken for other negative emotions, while happiness and surprise were most often mistaken for neutral emotion (see Table 2). In relative frequencies, fear was the affect individuals with FEP found most difficult to identify (38.3 % mistakes), followed by sadness (34.6 %), disgust (29 %), anger (25.4 %), happiness (17.5 %), surprise (7.5 %) and neutral (6 %). For controls, sadness was the most difficult emotion (31.1 % mistakes), followed by fear (27.7 %), happiness (14 %), disgust (12.8 %), anger (10.2 %), surprise (3.8 %), and neutral (no mistakes).

**Table 2:** Separate affects with the emotions participants chose when their responses were incorrect.

Participants with FEP				Control Participants			
Emotion Correct/Total % mistakes	Mistaken for	%	Frequency	Emotion Correct/Total % mistakes	Mistaken for	%	Frequency
**Anger**	Disgust	61.2	52	**Anger**	Disgust	59.3	16
85/335	Neutral	10.6	9	27/265	Fear	19.0	5
25.4%	Fear	10.6	9	10.2 %	Neutral	11.1	3
	Surprise	8.2	7		Sadness	7.4	2
	Sadness	5.9	5		Happiness	3.7	1
	Happiness	0.0	0		Surprise	0.0	0
	NA	3.5	3		NA	0.0	0
**Disgust**	Anger	49.5	48	**Disgust**	Anger	47.1	16
97/335	Sadness	12.4	12	34/265	Sadness	26.5	9
29.0 %	Neutral	10.3	10	12.8 %	Fear	14.7	5
	Surprise	10.3	10		Surprise	11.8	4
	Fear	7.2	7		Happiness	0.0	0
	Happiness	2.1	2		Neutral	0.0	0
	NA	8.2	8		NA	0.0	0
**Fear**	Surprise	65.0	50	**Fear**	Surprise	59.1	26
77/201	Disgust	18.2	14	44/159	Disgust	31.8	14
38.3 %	Neutral	6.5	5	27.7 %	Sadness	4.5	2
	Sadness	2.6	2		Anger	2.3	1
	Happiness	2.6	2		Happiness	0.0	0
	Anger	2.6	2		Neutral	0.0	0
	NA	2.6	2		NA	2.3	1
**Happiness**	Neutral	73.2	60	**Happiness**	Neutral	86.5	45
82/469	Surprise	20.7	17	52/371	Surprise	13.5	7
17.5 %	Fear	1.2	1	14.0 %	Fear	0.0	0
	Disgust	1.2	1		Disgust	0.0	0
	Sadness	1.2	1		Sadness	0.0	0
	Anger	0.0	0		Anger	0.0	0
	NA	2.4	2		NA	0.0	0
**Neutral**	Fear	31.3	5	**Neutral**	Surprise	0.0	0
16/268	Surprise	25.0	4	0/212	Fear	0.0	0
6.0 %	Disgust	12.5	2	0.0 %	Disgust	0.0	0
	Sadness	12.5	2		Happiness	0.0	0
	Happiness	12.5	2		Sadness	0.0	0
	Anger	6.3	1		Anger	0.0	0
	NA	0.0	0		NA	0.0	0
**Sadness**	Neutral	61.9	86	**Sadness**	Neutral	65.7	65
139/402	Disgust	16.6	23	99/318	Disgust	15.2	15
34.6 %	Fear	8.6	12	31.1 %	Fear	13.1	13
	Anger	7.2	10		Surprise	4.0	4
	Surprise	2.9	4		Anger	1.0	1
	Happiness	0.7	1		Happiness	1.0	1
	NA	2.2	3		NA	0.0	0
**Surprise**	Neutral	50.0	10	**Surprise**	Neutral	37.5	3
20/268	Happiness	15.0	3	8/212	Happiness	37.5	3
7.5 %	Fear	15.0	3	3.8 %	Fear	12.5	1
	Anger	5.0	1		Anger	12.5	1
	Disgust	5.0	1		Disgust	0.0	0
	Sadness	5.0	1		Sadness	0.0	0
	NA	5.0	1		NA	0.0	0

No statistically significant partial correlation was detected between FEFA-2 scores and PANSS positive or PANSS negative in the FEP group (see Table 3). This remained true regardless of the correlation analysis method used.

**Table 3:** Associations between psychotic symptoms and FAR in FEP, using Kendall's tau partial correlation with age as a covariate.

	FEFA-2 Total		FEFA-2 Negative		FEFA-2 Happiness	
	τ	*p*	τ	*p*	τ	*p*
PANSS Positive	-.045	0.595	-.088	0.296	.071	0.397
PANSS Negative	-.036	0.669	-.086	0.306	-.018	0.831

As a robustness check, all analyses were re-run with the excluded outlier, and it did not change the results.

### 3.2 Exploratory analyses

ANCOVAs were also run for every “pure” emotion separately (see Table 4). For each affect, the confidence intervals suggest that individuals with FEP, in general, perform somewhat worse than control participants. The estimates range between zero difference (surprise), negligible differences (sadness, fear), to relatively large group differences for disgust and anger, where individuals with FEP in general score half a point lower than controls.

**Table 4:** Exploratory analysis FAR of surprise, disgust, anger, fear, sadness and neutral, uncorrected p-values.

Affect	Beta (bootstrapped 95 % conf)	p-value (bootstrapped)	Effect size (Cohen's *d*)
Surprise	0.000 (-0.33/0.04)	0.152	0.26
Disgust	-0.481 (-1.17/-0.41)	<0.001	0.66
Anger	-0.662 (-1.12/-0.43)	<0.001	0.73
Fear	-0.361 (-0.64/-0.01)	0.043	0.37
Sadness	-0.132 (-0.65/0.17)	0.291	0.20
Neutral∗	NA	NA	NA

∗ Neutral emotion was not computed as the control group had no variance.

Group differences between individuals with FEP and controls on the FEFA Mixed category were (Beta = -0.92 [95 % conf: -1.63/-0.63], bootstrapped p < 0.001, Cohen's d = 0.78), indicating that in general individuals with FEP scored one point lower than controls.

Partial correlations between the FEFA Mixed category and PANSS subscales and items were negative, with effect sizes ranging from near zero (PANSS Hallucination, τ = -.035) to weak (PANSS Delusions, τ = -.203, PANSS Positive, τ = -.157, PANSS Negative, τ = -.119). No clear pattern was seen in the partial correlations between FEFA Negative/Total and PANSS items, and effect sizes were small (τ = -.101 to +.112).

Exploratory analyses comparing anti-psychotic drug-naïve individuals with FEP to medicated individuals with FEP and control participants suggest that, drug-naïve participants performed slightly better than medicated participants in general FAR ability and FAR of negative emotions (FEFA Total: Beta = -1.72 [95 % conf -3.39/-0.21], p = 0.031; FEFA Negative: Beta = -0.75 [95 % conf -2.80/-0.15], p = 0.035) but were still impaired compared to controls (FEFA Total: Beta = 1.42 [95 % conf 0.55/3.10], p = 0.009; FEFA Negative: 1.46 [95 % conf 0.36/2.50], p = 0.013). In FAR of positive affect, drug-naïve participants with FEP performed slightly better than medicated participants with FEP (FEFA Happiness: Beta = -0.50 [95 % conf -0.97/-0.12], p = 0.011), and at the same level as controls (FEFA Happiness: Beta = -0.01 [95 % conf -0.46/0.29], p = 0.644). However, these exploratory results, not corrected for multiple comparison, changed in the robustness check when the outlier was included and should therefore be regarded as preliminary. Drug naïve and medicating participants were largely similar regarding age, sex, diagnosis, and symptom severity of psychosis, but more drug naïve participants were hospitalized compared to those who medicated (n = 20 vs 13).

## 4 Discussion

In this study, we investigated the FAR ability in antipsychotic drug naïve or briefly medicated individuals with FEP and healthy control participants. Our findings are in line with some earlier studies (Allott et al., 2015); Barkl et al., 2014; Bosnjak et al., 2019; Catalan et al., 2016; Healey et al., 2016; Tripoli et al., 2022) showing that individuals with FEP performed worse than controls in general FAR and FAR of negative affect. This has also been consistently reported in individuals with schizophrenia (Chan et al., 2010; Mote and Kring, 2016). The exploratory analyses revealed that these group differences mainly were driven by impaired recognition of anger and disgust. Our findings provide additional support for the presence of these difficulties in the early stages of psychotic disorders, also in drug naïve patients and those with a short exposure to antipsychotic medication.

In line with previous research on FEP (Edwards et al., 2001; Healey et al., 2016; Leung et al., 2011) and schizophrenia (Mitrovic et al., 2020), there was no significant difference in FAR of happiness between individuals with FEP and controls. Also, in line with some previous studies on FEP (Allott et al., 2015; Thompson et al., 2012), but contrary to others both on FEP (Bliksted et al., 2017; Won et al., 2019) and schizophrenia (Kohler et al., 2010), we failed to find any significant association between FAR and symptoms of psychosis. This may indicate that FAR deficits in individuals with FEP do not underlie either psychotic symptoms such as hallucinations or delusions or negative symptoms such as decreased interest or lack of motivation. These discrepancies in findings may however be explained by methodological differences between studies, such as the use of different tests and tasks to measure FAR. For example, some studies that found associations to psychotic symptoms utilized tests of emotion intensity (where participants are asked to pick between happy, sad, and neutral and grade the intensity of happiness and sadness) instead of emotion labeling (Daros et al., 2014; Herbener et al., 2005; Yang et al., 2015). Other studies that found associations used tests where FAR items were only presented for 400–1000 ms (Edwards et al., 2001; Romero-Ferreiro et al., 2016; Won et al., 2019; Yang et al., 2015), a fraction of the time allowed in FEFA-2. This requires additional abilities besides FAR, such as processing speed and working memory capacity, which could explain their results. Additionally, some studies that found associations do not report if corrections for multiple comparisons were made (Edwards et al., 2001; Lee et al., 2015; Liu et al., 2019; Romero-Ferreiro et al., 2016; Won et al., 2019), potentially limiting the conclusions that can be drawn. In our sample, both FEP and control participants primarily mistook negative affects for other negative affects, while surprise and happiness were primarily mistaken for neutral affect. This lack of bizarre choices may suggest that individuals with FEP do not make idiosyncratic interpretations of facial expressions, and could possibly be viewed as additional support against a relationship between psychotic symptoms and FAR. However, participants in the current study had moderate symptoms of psychosis, and therefore these results might not be generalized to more severe cases.

### 4.1 Strengths and limitations

Strengths of this study include the pre-registration of the statistical analysis, a relatively short period of untreated psychosis at inclusion, and all included individuals were thoroughly assessed regarding psychiatric symptoms close in time to the FAR testing. In addition, individuals with FEP were either drug naïve or had short exposure to antipsychotic medication.

A possible limitation is the lack of exclusion of comorbid psychiatric diagnoses which might have affected our results. However, psychiatric comorbidity in individuals with FEP is quite common, and therefore stricter exclusion criteria would have limited both recruitment and the generalizability of our findings. It cannot be excluded that differences in FAR performance were due to differences in global cognitive ability. However, if there is an effect of global cognition, a recent meta-analysis suggests that it should be fairly modest (Schlegel et al., 2020) and it is unlikely that it explains the group differences we observed. In addition, included participants had moderate symptoms of psychosis making it difficult to generalize our findings to individuals with more severe psychotic symptoms. However, severely ill participants might not be able to make an informed consent, or for example, concentrate during FAR tasks. Further, as our control-group was recruited on an online platform often used by students, we cannot exclude a higher educational attainment in the control group.

### 4.2 Clinical implication

The observed impairments in FAR in individuals with FEP suggest that it could be beneficial to work towards a more flexible interpretation of others’ emotions during psychotherapy, in the early stages of psychotic disorders. The fact that individuals with FEP easily recognize positive affects is encouraging, given that therapists who genuinely display positive affects combined with a friendly manner seem to increase psychotherapy outcomes (Bourke et al., 2021; Harper Romeo et al., 2014). Training of FAR early in psychotic disorders might also be beneficial.

## 5 Conclusion

In the present study, we found that drug naïve or briefly medicated individuals with FEP, displayed significantly impaired general FAR and FAR of negative affects compared to controls, indicating a disability early in the process of psychotic disorders. No group differences in the recognition of happiness were found. All the earlier mentioned results are in line with previous findings in FEP and schizophrenia. No association between FAR and symptoms of psychosis was found, contrary to findings in individuals with schizophrenia. Exploratory analyses in our patient sample revealed that the group differences in general FAR and FAR of negative affect were mainly driven by impaired FAR of anger and disgust. The pattern of mistakes, made by patients and controls, was similar, indicating that individuals with FEP do not make idiosyncratic interpretations of facial affective expressions.

## Declarations

### Author contribution statement

Cornelia Larsson, Maria Lee: Conceived and designed the experiments; Analyzed and interpreted the data; Wrote the paper.

Tobias Lundgren: Analyzed and interpreted the data; Wrote the paper.

Sophie Erhart, Carl M Cellgren, Simon Cervenka: Conceived and designed the experiments; Contributed reagents, materials, analysis tools or data; Wrote the paper.

Jaqueline Borg, Sven Bölte: Contributed reagents, materials, analysis tools or data; Wrote the paper.

Helena Fatouros-Bergman: Conceived and designed the experiments; Performed the experiments; Analyzed and interpreted the data; Wrote the paper.

### Funding statement

The study was supported by the Swedish Research Council (Grant No. 523-2014-3467 (SC), 2017-00875 (SE)), Karolinska Institutet and Stockholm County Council (20160328, 20180487 [SC], (20190175) [SE], 20190447 [CMS]) Torsten Söderberg Stiftelse (SE), and Centre for Psychiatric Research (Award No. CPF 100/2011 [HFB]). None of the funding sources had any involvement in the study.

### Data availability statement

Due to institutional restrictions, data cannot be shared openly but can be made available upon request on a case-by-case basis as allowed by the legislation and ethical permits. Requests for access can be made to the Karolinska Institutet's Research Data Office at rdo@ki.se.

### Declaration of interest's statement

The authors declare the following conflict of interests: Sven Bölte was an author, consultant, or lecturer for Medice and Roche during the last 3 years. He receives royalties for textbooks and diagnostic tools from Hogrefe Publishers and is a shareholder in SB Education and Psychological Consulting and NeuroSupportSolutions International. Carl M. Sellgren is a scientific advisor to Outermost Therapeutics Inc.

### Additional information

No additional information is available for this paper.
